# Supplementary material for: Mechanochemical active ratchet
Source: Sci Rep. 2023 Nov 23;13:20572. doi: 10.1038/s41598-023-47465-2 (PMC10667355; doi:10.1038/s41598-023-47465-2)
Supplement: Supplementary file 1 — Supplementary Information. [file 41598_2023_47465_MOESM1_ESM.pdf]

Supplementary Information: Mechanochemical Active Ratchet

Artem Ryabov[1,2,3] and Mykola Tasinkevych[2,3,4,5]

[1]*Charles University, Faculty of Mathematics and Physics,*
Department of Macromolecular Physics, V Holešovičkách 2, CZ-18000 Praha 8, Czech Republic

[2]*Departamento de Física, Faculdade de Ciências,*
Universidade de Lisboa, 1749-016 Lisboa, Portugal

[3]*Centro de Física Teórica e Computacional, Faculdade de Ciências,*
Universidade de Lisboa, 1749-016 Lisboa, Portugal

[4]*SOFT Group, School of Science and Technology,*
Nottingham Trent University, Clifton Lane,
Nottingham NG11 8NS, United Kingdom

[5]*International Institute for Sustainability with Knotted Chiral Meta Matter,*
Hiroshima University, Higashihiroshima, Japan.

(Dated: November 10, 2023)

This Supplemental Information provides additional technical details about Brownian dynamics simulations that were used to obtain the results presented in the main text. Also, the simplified approximate Langevin equation is derived.

Supplementary Note 1: Langevin Equations in Dimensionless Units

We consider an active nanoparticle diffusing in the force field

$$\boldsymbol{F}(\boldsymbol{r}) = (F(x), f_\perp), \tag{SM1}$$

with the constant y-component $f_\perp$ and the x-component being

$$F(x) = f_\parallel - \frac{\mathrm{d}V}{\mathrm{d}x} = f_\parallel - \frac{\pi V_0}{\lambda}\left[2\cos\left(\frac{2\pi x}{\lambda}\right) + \cos\left(\frac{4\pi x}{\lambda}\right)\right], \tag{SM2}$$

where $V(x) = V(x+\lambda)$ is the external λ-periodic potential. The system of Langevin equations governing dynamics of the particle's center of mass position $\boldsymbol{r}(t) = (x(t), y(t))$, and the orientation $\phi(t)$ is

$$\frac{\mathrm{d}x}{\mathrm{d}t} = v_\mathrm{a}(x,t)\cos\phi(t) + \mu F(x) + \sqrt{2D}\,\xi_x(t), \tag{SM3a}$$

$$\frac{\mathrm{d}y}{\mathrm{d}t} = v_\mathrm{a}(x,t)\sin\phi(t) + \mu f_\perp + \sqrt{2D}\,\xi_y(t), \tag{SM3b}$$

$$\frac{\mathrm{d}\phi}{\mathrm{d}t} = \sqrt{2D_\mathrm{r}}\,\xi_\mathrm{r}(t), \tag{SM3c}$$

where

$$v_{\mathrm{a}}(x,t) = u + \mu_{\mathrm{c}}\left[F(x)\cos\phi(t) + f_{\perp}\sin\phi(t)\right] + \sqrt{2D_{\mathrm{c}}}\,\xi_{\mathrm{c}}(t), \tag{SM4}$$

is the active speed and the Gaussian white noise processes $\xi_{\alpha}(t)$ satisfy

$$\langle \xi_{\alpha}(t) \rangle = 0, \tag{SM5}$$

$$\langle \xi_{\alpha}(t)\xi_{\beta}(t') \rangle = \delta_{\alpha\beta}\delta(t-t'), \tag{SM6}$$

$\alpha, \beta \in \{x, y, \mathrm{r}, \mathrm{c}\}$.

As the natural unit of length we choose the period λ of the external potential $V(x)$. (Alternatively, one could use the hydrodynamic radius of the nanoparticle.) As the natural unit of time we adopt the characteristic timescale of the fastest process in the model: the rotational diffusion. This characteristic timescale is given by $1/D_{\mathrm{r}}$. Expressing coordinates and time in these units yields

$$X = \frac{x}{\lambda}, \tag{SM7}$$

$$Y = \frac{y}{\lambda}, \tag{SM8}$$

$$\tau = D_{\mathrm{r}}t. \tag{SM9}$$

Let us now derive the Langevin equations for the dimensionless coordinates X and Y with respect to the time τ. These equations will be formulated by means of dimensionless forces, dimensionless noises, and the dimensionless active speed.

The dimensionless x-component of the force $\bar{F}(X)$, the dimensionless constant x-component $\bar{f}_{\parallel}$, and the dimensionless y-component $\bar{f}_{\perp}$ are respectively defined as

$$\bar{F}(X) = \frac{\lambda F(\lambda X)}{k_{\mathrm{B}}T} = -\pi\bar{V}_0\left[2\cos(2\pi X) + \cos(4\pi X)\right] + \bar{f}_{\parallel}, \tag{SM10}$$

$$\bar{f}_{\parallel} = \frac{\lambda f_{\parallel}}{k_{\mathrm{B}}T}, \tag{SM11}$$

$$\bar{f}_{\perp} = \frac{\lambda f_{\perp}}{k_{\mathrm{B}}T}, \tag{SM12}$$

where the dimensionless amplitude of the periodic potential

$$\bar{V}_0 = \frac{V_0}{k_{\mathrm{B}}T} \tag{SM13}$$

is expressed in units of $k_{\mathrm{B}}T$.

Gaussian white noises are transformed according to

$$\zeta_{\alpha}(\tau) = \frac{1}{D_{\mathrm{r}}}\,\xi_{\alpha}\left(\frac{\tau}{D_{\mathrm{r}}}\right), \tag{SM14}$$

$\alpha \in \{x, y, \mathrm{r}, \mathrm{c}\}$. The transformed dimensionless processes $\zeta_\alpha(\tau)$ are independent, each of them has the mean value zero and they are delta-correlated in dimensionless time, similarly to the original noises $\xi_\alpha(t)$ satisfying Eqs. (SM5) and (SM6). Specifically, $\langle \zeta_\alpha(\tau) \rangle = 0$ follows from (SM5), and $\langle \zeta_\alpha(\tau)\zeta_\beta(\tau') \rangle = \delta_{\alpha\beta}\delta(\tau - \tau')$ is justified employing the identity $\delta(\tau/D_\mathrm{r}) = D_\mathrm{r}\delta(\tau)$.

In course of the transition to the dimensionless units, the diffusion coefficients D and D_c are scaled by the factor $\lambda^2 D_\mathrm{r}$ giving two dimensionless quantities

$$\bar{D} = \frac{D}{\lambda^2 D_\mathrm{r}}, \tag{SM15}$$

$$\bar{D}_\mathrm{c} = \frac{D_\mathrm{c}}{\lambda^2 D_\mathrm{r}}. \tag{SM16}$$

Furthermore, the combination λD_r sets the unit of velocity, yielding

$$\bar{v}_\mathrm{a}(X, \tau) = \frac{v_\mathrm{a}(\lambda X, \tau/D_\mathrm{r})}{\lambda D_\mathrm{r}} = \bar{u} + \bar{D}_\mathrm{c}\left[\bar{F}(X)\cos\phi(\tau) + \bar{f}_\perp \sin\phi(\tau)\right] + \sqrt{2\bar{D}_\mathrm{c}}\,\zeta_\mathrm{c}(\tau), \tag{SM17}$$

$$\bar{u} = \frac{u}{\lambda D_\mathrm{r}}. \tag{SM18}$$

The Langevin equations (SM3), when expressed using these dimensionless quantities, read

$$\frac{\mathrm{d}X}{\mathrm{d}\tau} = \bar{v}_\mathrm{a}(X, \tau)\cos\phi(\tau) + \bar{D}\bar{F}(X) + \sqrt{2\bar{D}}\,\zeta_x(\tau), \tag{SM19a}$$

$$\frac{\mathrm{d}Y}{\mathrm{d}\tau} = \bar{v}_\mathrm{a}(X, \tau)\sin\phi(\tau) + \bar{D}\bar{f}_\perp + \sqrt{2\bar{D}}\,\zeta_y(\tau), \tag{SM19b}$$

$$\frac{\mathrm{d}\phi}{\mathrm{d}\tau} = \sqrt{2}\,\zeta_\mathrm{r}(\tau). \tag{SM19c}$$

Numerical values of the dimensionless model parameters $\bar{D}$, $\bar{D}_\mathrm{c}$, and $\bar{u}$, defined in Eqs. (SM15), (SM16), and (SM18), follow from Eqs. (6)-(10) in the main text:

$$\bar{D} = 0.03, \tag{SM20}$$

$$\bar{D}_\mathrm{c} \approx 0.0022, \tag{SM21}$$

$$\bar{u} \approx 1.7 \times 10^{-6}. \tag{SM22}$$

Supplementary Note 2: Brownian Dynamics Simulations

In our numerical simulations, we obtained approximate solutions of Eqs. (SM19) by implementing the Euler–Maruyama method [1]. Having values of $X(\tau)$, $Y(\tau)$, and $\phi(\tau)$, the method approximates the corresponding values of $X(\tau + \Delta\tau)$, $Y(\tau + \Delta\tau)$, and $\phi(\tau + \Delta\tau)$ according to the recurrence

relations

$$X(\tau+\Delta\tau) = X(\tau) + \left[\bar{v}_{\rm a}(X(\tau),\tau)\cos\phi(\tau) + \bar{D}\bar{F}(X)\right]\Delta\tau + \sqrt{2\bar{D}\Delta\tau}\,\mathcal{N}_x(0,1), \tag{SM23a}$$

$$Y(\tau+\Delta\tau) = Y(\tau) + \left[\bar{v}_{\rm a}(X(\tau),\tau)\sin\phi(\tau) + \bar{D}\bar{f}_\perp\right]\Delta\tau + \sqrt{2\bar{D}\Delta\tau}\,\mathcal{N}_y(0,1), \tag{SM23b}$$

$$\phi(\tau+\Delta\tau) = \phi(\tau) + \sqrt{2\Delta\tau}\,\mathcal{N}_{\rm r}(0,1), \tag{SM23c}$$

where

$$v_{\rm a}(X(\tau),\tau) = \bar{u} + \bar{D}_{\rm c}\left[\bar{F}(X(\tau))\cos\phi(\tau) + \bar{f}_\perp\sin\phi(\tau)\right] + \sqrt{\frac{2\bar{D}_{\rm c}}{\Delta\tau}}\,\mathcal{N}_{\rm c}(0,1), \tag{SM24}$$

and $\mathcal{N}_\alpha(0,1)$, $\alpha \in \{x, y, {\rm r}, {\rm c}\}$, are independent and identically distributed random numbers drawn from the normal distribution with zero mean and unit variance.

In all simulations, at the initial time $\tau = 0$, we set $X(0) = Y(0) = 0$. The initial particle orientation is assumed to be random, i.e., $\phi(0)$ is a random variable homogeneously distributed within the interval $[0, 2\pi)$.

Since $\bar{D}$ and $\bar{D}_{\rm c}$, and $\bar{u}$ are much smaller than one, see Eqs. (SM20)-(SM22), the translational diffusion and the active motion take place on significantly slower timescales than the rotational diffusion does. Therefore, to achieve a satisfactory precision of the numerical scheme (SM23), it is necessary to choose $\Delta\tau$ such that the rotational diffusion process is simulated precisely enough. The difference in time scales then would ensure that the particle displacements caused by the two slow processes are approximated well too.

We have found that $\Delta\tau = 10^{-3}$, used to generate all simulation results displayed in the main text, is small enough for this purpose. To verify that the results of simulations do not depend on $\Delta\tau$, we performed simulations for a few selected parameter sets and $\Delta\tau = 10^{-4}$ and $\Delta\tau = 10^{-5}$.

As a consequence of the timescale separation in our model, long overall simulation time $\tau_{\rm max}$ (compared to $\Delta\tau$) must be chosen to generate a noticeable translational motion of the particle across several wells of the potential $V(x)$. The long $\tau_{\rm max}$ is also necessary to eliminate all transient effects. We have used $\tau_{\rm max} = 10^3$.

For each given set of model parameters, the mean particle velocity $\langle v_x \rangle$ in the x-direction has been obtained as the long-time average of the particle displacement along the x-axis:

$$\langle v_x \rangle = \frac{1}{N_{\rm tr}} \sum_{i=1}^{N_{\rm tr}} \frac{x_i(\tau_{\rm max})}{\tau_{\rm max}}, \tag{SM25}$$

where $x_i(\tau_{\rm max})$ corresponds to the final point of the i-th trajectory and $N_{\rm tr}$ is the total number of trajectories. For all reported data points, we used $N_{\rm tr} = 10^4$. We have checked that increasing the

value of $N_{\rm tr}$ to 10^5 and 10^6 (and of $\tau_{\max}$ to $\tau_{\max} = 10^4$ and 10^5) does not affect the results. These stability checks were performed for larger values of $\bar{V}_0$ and $\bar{f}_\perp$ where larger numerical errors are expected to occur.

Supplementary Note 3: Approximate Langevin Equation for the Longitudinal Coordinate

The Langevin equation (SM3a) for the longitudinal coordinate x is given by

$$\frac{\mathrm{d}x}{\mathrm{d}t} = \left\{ u + \mu_{\rm c} \left[F(x) \cos\phi(t) + f_\perp \sin\phi(t) \right] + \sqrt{2D_{\rm c}}\, \xi_{\rm c}(t) \right\} \cos\phi(t) + \mu F(x) + \sqrt{2D}\, \xi_x(t), \quad \text{(SM26)}$$

which we can rearrange to a compact form

$$\frac{\mathrm{d}x}{\mathrm{d}t} = u\cos\phi(t) + \frac{\mu_{\rm c} f_\perp}{2} \sin[2\phi(t)] + \left[\mu + \mu_{\rm c}\cos^2\phi(t)\right] F(x) + \sqrt{2k_{\rm B}T \left[\mu + \mu_{\rm c}\cos^2\phi(t)\right]}\, \xi(t), \quad \text{(SM27)}$$

where $\xi(t)$ is the Gaussian white noise process satisfying $\langle \xi(t) \rangle = 0$ and $\langle \xi(t)\xi(t') \rangle = \delta(t-t')$. Equation (SM27) is equivalent to Eq. (SM26). In deriving Eq. (SM27) from Eq. (SM26), we have employed the identity $\sin(2\phi) = 2\sin\phi\cos\phi$, the fluctuation-dissipation theorems $D = \mu k_{\rm B}T$, $D_{\rm c} = \mu_{\rm c} k_{\rm B} T$ from the main text, and the property of Gaussian random variables [2]:

$$\sqrt{\sigma_1^2}\, \mathcal{N}_1(0,1) + \sqrt{\sigma_2^2}\, \mathcal{N}_2(0,1) = \sqrt{\sigma_1^2 + \sigma_2^2}\, \mathcal{N}_3(0,1), \quad \text{(SM28)}$$

where $\mathcal{N}_\alpha(0,1)$, $\alpha \in \{1,2,3\}$, are independent and identically distributed normal random variables with zero mean and unit variance.

For the values of parameters u, μ, $\mu_{\rm c}$, $f_\perp$, and $D_{\rm r}$ used in the main text, Eq. (SM27) can be considerably simplified. This is done in two steps.

First, for $u \lesssim 1000$ nm/s we can neglect the term proportional to u in Eq. (SM27). This approximation is valid for active particles with smaller hydrodynamic radii $R_{\rm h}$. It results from the large value of $D_{\rm r}$, $D_{\rm r} \sim 1/R_{\rm h}^3$, Eq. (7) in the main text. Thus, for $u \approx 10$ nm/s, used in all figures in the main text, Eq. (SM27) reduces to

$$\frac{\mathrm{d}x}{\mathrm{d}t} \approx \frac{\mu_{\rm c} f_\perp}{2} \sin[2\phi(t)] + \left[\mu + \mu_{\rm c}\cos^2\phi(t)\right] F(x) + \sqrt{2k_{\rm B}T \left[\mu + \mu_{\rm c}\cos^2\phi(t)\right]}\, \xi(t). \quad \text{(SM29)}$$

Second, according to Eqs. (6) and (8), $D_{\rm c}/D = \mu_{\rm c}/\mu \approx 0.073$, i.e., in the expression $\left[\mu + \mu_{\rm c}\cos^2\phi(t)\right]$, the term $\mu_{\rm c}\cos^2\phi(t)$ is negligible as compared to μ. Numerically we have found, that the results obtained after the approximation

$$\mu + \mu_{\rm c}\cos^2\phi(t) \approx \mu + \frac{\mu_{\rm c}}{2}, \quad \text{(SM30)}$$

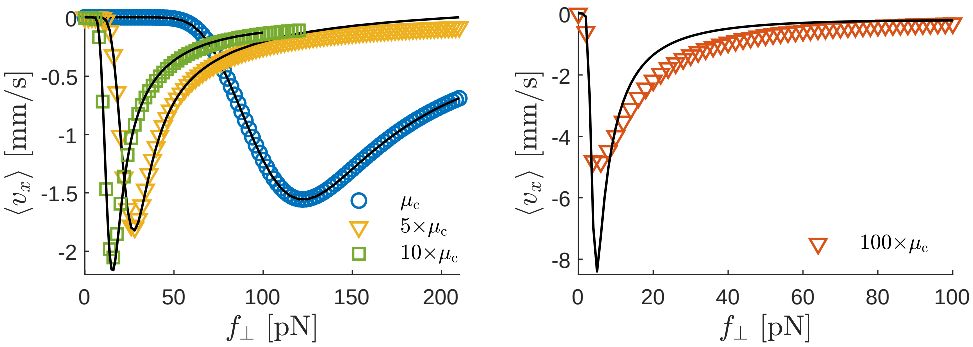

FIG. S1. Mean velocity $\langle v_x \rangle$ in the x-direction, Eq. (SM25), for $f_\parallel = 0$, $V_0 = 20\, k_B T$, and four values of the parameter μ_c. Other model parameters are the same as in Figs. 2 and 3 in the main text. Symbols (lines) represent results based on the numerical integration of the exact Eq. (SM26) [approximate Eq. (SM31)]. Left panel: circles mark results for the reference value of μ_c obtained from Eq. (8) in the main text, triangles (squares) correspond to values of μ_c five (ten) times higher than the reference value. Right panel: μ_c hundred times higher than the reference value.

i.e., when we approximate the nonnegative function by its average, $\cos^2 \phi(t) \approx 1/2$, fit the simulated data slightly better than the curves obtained by the simple approximation $\left[\mu + \mu_c \cos^2 \phi(t)\right] \approx \mu$, the difference being of the order of percents. Employing the approximation (SM30), gives us

$$\frac{\mathrm{d}x}{\mathrm{d}t} \approx \frac{\mu_c f_\perp}{2} \sin[2\phi(t)] + \left(\mu + \frac{\mu_c}{2}\right) F(x) + \sqrt{2 k_B T \left(\mu + \frac{\mu_c}{2}\right)}\, \xi(t). \tag{SM31}$$

Setting further $f_\parallel = 0$ yields Eq. (11) in the main text.

In the main text, the approximate Eq. (SM31) is used in the qualitative discussion of properties of the active ratchet effect. Even though no reported simulation results are based on the solution of this equation, let us now test its validity by comparing the mean velocities $\langle v_x \rangle$, Eq. (SM25), obtained by integrating the exact Eq. (SM26) and the approximate Eq. (SM31). Such a comparison is presented in Fig. S1 showing $\langle v_x \rangle$ as a function of $f_\perp$ for four μ_c. In the left panel, we have used the reference value of μ_c obtained from Eq. (8) in the main text and increased this reference μ_c by 5, 10, and 100 times. The right panel shows the extreme case of 100 times reference μ_c. Symbols (lines) represent results based on Eq. (SM26) [Eq. (SM31)]. In the left panel, Eq. (SM31) approximates $\langle v_x \rangle$ reasonably well. In the right panel, significant quantitative deviations between the mean velocities in the two models occur. Despite this quantitative disagreement, the overall shape of the velocity-force relations is similar. Also, both curves have a minimum at comparable values of perpendicular forces.

Supplementary References

[1] P. E. Kloeden and E. Platen, *Numerical Solution of Stochastic Differential Equations*, Stochastic Modelling and Applied Probability (Springer Berlin, Heidelberg, 1992).

[2] D. E. Lemons, *An Introduction to Stochastic Processes in Physics*, Stochastic Modelling and Applied Probability (Johns Hopkins University Press, Baltimore, MD, USA, 2002).
